# Avocado Seeds-Mediated Alleviation of Cyclosporine A-Induced Hepatotoxicity Involves the Inhibition of Oxidative Stress and Proapoptotic Endoplasmic Reticulum Stress

**DOI:** 10.3390/molecules27227859

**Published:** 2022-11-14

**Authors:** Mohammed A. El-Magd, Amina M. G. Zedan, Nahla S. Zidan, Mohamed I. Sakran, Omar Bahattab, Atif Abdulwahab A. Oyouni, Osama M. Al-Amer, Adel I. Alalawy, Amira M. Elmoslemany

**Affiliations:** 1 Department of Anatomy, Faculty of Veterinary Medicine, Kafrelsheikh University, Kafrelsheikh 33516, Egypt; 2 Biological and Environmental Sciences Department, Faculty of Home Economic, Al Azhar University, Tanta 31732, Egypt; 3 Department of Nutrition and Food Science, Faculty of Home Economics, Tabuk University, Tabuk 47512, Saudi Arabia; 4 Department of Home Economics, Faculty of Specific Education, Kafrelsheikh University, Kafrelsheikh 33516, Egypt; 5 Department of Biochemistry, Faculty of Sciences, University of Tabuk, Tabuk 47512, Saudi Arabia; 6 Biochemistry Division, Chemistry Department, Faculty of Science, Tanta University, Tanta 31512, Egypt; 7 Biology Department, Faculty of Sciences, University of Tabuk, Tabuk 47512, Saudi Arabia; 8 Genome and Biotechnology Unit, Faculty of Sciences, University of Tabuk, Tabuk 47512, Saudi Arabia; 9 Department of Medical Laboratory Technology, Faculty of Applied Medical Sciences, University of Tabuk, Tabuk 47512, Saudi Arabia; 10 Nutrition and Food Science Department, Faculty of Home Economic, Al Azhar University, Tanta 31732, Egypt

**Keywords:** avocado seeds, cyclosporine A, hepatotoxicity, oxidative stress, ER stress

## Abstract

Previous studies reported disrupted hepatic function and structure following the administration of cyclosporine A (CsA) in humans and animals. Recently, we found that avocado seeds (AvS) ameliorated CsA-induced nephrotoxicity in rats. As a continuation, herein we checked whether AvS could also attenuate CsA-induced hepatotoxicity in rats. Subcutaneous injection of CsA (5 mg/kg) for 7 days triggered hepatotoxicity in rats, as indicated by liver dysfunction, redox imbalance, and histopathological changes. Oral administration of 5% AvS powder for 4 weeks ameliorated CsA-induced hepatotoxicity, as evidenced by (1) decreased levels of liver damage parameters (alanine aminotransferase (ALT), aspartate aminotransferase (AST), alkaline phosphatase (ALP), and bilirubin), (2) resumed redox balance in the liver (reduced malondialdehyde (MDA) and increased superoxide dismutase (SOD), catalase (CAT), and glutathione peroxidase (GPx)), (3) downregulated hepatic expression of endoplasmic reticulum (ER) stress-related genes (X-box binding protein 1 (*XBP1*), binding immunoglobulin protein (*BIP*), C/EBP homologous protein (*CHOP*)), and apoptosis-related genes (*Bax* and *Casp3*), (4) upregulated expression of the anti-apoptotic gene *Bcl2*, (5) reduced DNA damage, and (6) improved liver histology. These results highlight the ability of AvS to ameliorate CsA-induced hepatotoxicity via the inhibition of oxidative stress and proapoptotic ER stress.

## 1. Introduction

Liver damage caused by hepatotoxicity is considered a serious global health concern. Among different causes of hepatotoxicity, drug-induced hepatotoxicity is more serious and limits drug usage [1,2]. Consequently, the therapy and prevention of drug-induced hepatotoxicity has become an urgent demand. Cyclosporine A (CsA) is one of the two common calcineurin inhibitor drugs used effectively to suppress immunity after organ transplantation surgery [3]. However, patients should be injected with CsA for a very long time to avoid an autoimmune response. Unfortunately, this long administration causes many health problems, especially nephrotoxicity [4] and hepatotoxicity [5]. Several studies on animals reported that the administration of CsA disturbed liver function and structure [6,7,8,9,10,11,12,13,14]. Oxidative stress, inflammation, and apoptosis are three main mechanisms that have been shown to be involved in CsA-mediated hepatotoxicity [6,7,9,10,11,12,14,15]. CsA can also increase DNA damage and fragmentation in renal cells and sperm [10,16]. Activation of endoplasmic reticulum (ER) stress is another recently researched mechanism by which CsA could exert its nephrotoxic effect [17]. The ER stress pathway involves many genes such as cleavage of X-box binding protein 1 (*XBP1*), binding immunoglobulin protein (*BIP*), and C/EBP homologous protein (*CHOP*) [18]. However, to date, little is known about whether CsA-induced hepatotoxicity is also mediated by ER stress activation.

Several previous studies reported the notable ameliorative effects of antioxidants on CsA-induced hepatotoxicity in lab animals [6,9,10,11,12,13,14,19,20,21,22]. Avocado (*Persea americana* Mill) is a plant whose seeds (AvS) and pulps have many health-promoting antioxidant constituents [23]. The potent antioxidant effect of AvS was attributed to high levels of flavonoids and phenolic compounds in these seeds [10,24,25]. Natural products and their flavonoids and phenols have been shown to abate hepatotoxicity and associated liver injury [2,26,27]. Moreover, AvS improved liver glycogen content in rats [28] and avocado pulp enhanced the liver function and structure deteriorated by potassium dichromate in the rat model of liver injury [29]. *P. americana* extracts also protected the liver against the toxic effect of CCl_4_ [30].

Recently, we reported that rats treated with AvS, which had potent antioxidant properties, abated the nephrotoxicity triggered by CsA [10]. However, the effect of AvS on CsA-induced hepatotoxicity has not yet been addressed. It is also worth knowing whether CsA-induced hepatotoxicity involves ER stress activation and whether this effect would be inhibited by AvS. Therefore, this study aimed to evaluate the effect of AvS on CsA-induced hepatotoxicity, with a special focus on oxidative stress, apoptosis, inflammation, and ER stress mechanisms of action. 

## 2. Materials and Methods 

### 2.1. Preparation of AvS Powder

Preparation of AvS was performed as previously detailed [10]. In brief, fresh *P. americana* fruits were bought from a local market in Kafrelsheikh, Egypt, and the seeds were obtained following avocado peeling. The seeds were hot-dried in an oven at 40 °C for 48 h until achieving a moisture level of 4%, crushed, and filtered in 40 mesh to obtain fine AvS powder.

### 2.2. Detection of AvS Phenolic Compounds by HPLC

Flavonoids and phenolic compounds were determined in the ethanolic extract of the AvS powder using an HPLC (Agilent Technologies 1100 series, Santa Clara, CA, USA) with a vehicle sampler, a diode array detector, an XDB-C18 analytical column, and a C18 guard column (Phenomenex, Torrance, CA, USA), as previously described [31]. The separation occurred using acetonitrile (solvent A) and acetic acid (solvent B). The injection volume was 20 μL, and the peaks were detected at three different wavelengths: 280, 320, and 360 nm. Peaks were compared with standards.

### 2.3. Experimental Design

Before starting the experiment, we got ethical approval (ID: KFS-127/85) from the Institutional Animal Care and Use Committee of Kafrelsheikh University in accordance with the guidelines of the Declaration of Helsinki. A total number of thirty-two albino Wistar male rats (160 ± 20 g) were housed in cages and fed a standard rat chow diet ad libitum with free access to water. Rats were acclimatized for 12 days under the optimal laboratory conditions (12 h light/12 h dark and 23 ± 2 °C), before the experiment was started. Animals were randomly allocated to four groups (*n* = 8/group). In the control (Cnt) group, rats were subcutaneously (SC) injected with 1 mL/kg body weight (BW) olive oil (vehicle) daily for the first week of the experiment. In the avocado seeds (AvS) group, 5% AvS powder (50 g AvS powder per Kg diet) was added to the basal diet for 4 weeks. In the CsA group, animals were SC injected with CsA at a concentration of 5 mg/kg BW daily in the first week. The dose and route of administration were chosen based on our previous study which showed no toxic effects for AvS [10]. CsA was purchased from Novartis Pharma Co., Plantation, FL, USA with a trading name of Sandimun Neoral^®^ in the form of capsules, and each capsule contained 25 mg CsA. Each capsule was dissolved in 5 mL olive oil and each rat was injected with 200 µL. In the combined group (CsA + AvS), rats were fed on a basal diet supplemented with 5% AvS powder for 4 weeks and SC injected with 5 mg/kg CsA daily in the first week. At the end of the experiment, blood samples were collected from the venous plexus of the medial canthus of the eyes in plain vacutainer tubes without anticoagulant. Rats were then euthanized by exsanguination and livers were quickly excised. 

### 2.4. Serum Biochemical Analysis 

Biochemical assays for liver damage parameters were performed on serum samples. To separate serum, blood samples were centrifuged at 3500× *g* for 15 min. The activities of aspartate transaminase (AST), alanine transaminase (ALT), and alkaline phosphatase (ALP) were quantified by kits purchased from Diamond-Diagnostics (Cairo, Egypt). Gamma glutamyl transpeptidase (γ-GTP), albumin, and bilirubin were measured by kits bought from BioMed-Diagnostics (EGY-CHEM, Cairo, Egypt).

### 2.5. Determination of Oxidant and Antioxidant Status in Liver

The levels of the lipid peroxidation marker MDA and the activities of the antioxidant enzymes (SOD, CAT, and GPx) were determined in the liver homogenate. To prepare this homogenate, 1 g liver specimen was homogenized in 9 mL ice-cold phosphate-buffered saline (PBS) and the mixture was centrifuged at 1000× *g* for 15 min at 4 °C. The levels of MDA and the activities of SOD, CAT, and GPx were determined in the supernatant. For quantification of MDA, the produced thiobarbituric acid-reactive substances (TBARS) were spectrophotometrically estimated at the absorbance of 530 nm, as previously detailed [32]. For quantification of SOD activities, the method of Misra and Fridovich [33] was applied, something which depends on the inhibition of epinephrine auto-oxidation into adrenochrome by SOD at pH 10.2. The method of Aebi [34] was used to spectrophotometrically measure CAT activities (as estimated by the degradation rate of H_2_O_2_) at 240 nm. GPx hepatic activities were estimated as previously detailed [35].

### 2.6. Real-Time PCR

Total RNA was extracted from liver specimens previously frozen at −80 °C using a Trizol reagent (Invitrogen, Carlsbad, CA, USA, Cat# 15596026). The concentration and purity of the isolated RNA were detected by a Q5000 Quawell nanodrop (USA), and RNA samples with considerable concentration and purity were reverse-transcribed into cDNA using the RevertAid H Minus Reverse (Thermo Scientific, Waltham, MA, USA, #EP04 51). The real-time PCR mix containing cDNA, Syber green master mix (2X Maxima, Thermo Scientific, # K0221, USA), and primers (Table 1) was put in StepOnePlus qPCR thermal cycler (Applied Biosystem, Foster City, CA, USA). Thermal conditions included one cycle of initial denaturation (94 °C/4 min), followed by 40 cycles of each denaturation (94 °C/40 s) and annealing extension (60 °C/1 min). The *GAPDH* was used as an internal control. Altered gene expression (fold change) was determined using the Livak method (2^−∆∆Ct^) relative to the control group, as previously detailed [36,37].

### 2.7. Comet Assay

DNA damage in the liver after various treatments was assessed by comet assay using GelRed stain and Komet 5 image analysis software (Kinetic Imaging, Ltd. Liverpool, UK), as previously described [10,38]. The extent of DNA damage in hepatic cells was quantified in 100 cells by a fluorescence microscope (at 40×). By this assay, we determined (1) damaged DNA (%) in the form of a comet that extended behind the intact circular DNA, (2) comet tail length (calculated from the center of the intact DNA to the end of the comet), and (3) tail moment.

### 2.8. Histopathology Examination

Liver specimens were fixed overnight in 10% formalin, embedded in paraffin, sectioned (5 µm), and stained with hematoxylin and eosin. The liver damage score [no lesion (0), mild (1), moderate (2), and severe (3)] was determined based on previous studies [2,11]. The scored histopathological lesions (dilation of hepatic sinusoids, mononuclear inflammatory cell infiltration, and congestion of central and portal veins) were examined in 10 fields/slide (at 40×) for 3 samples/group. 

### 2.9. Statistical Analysis

Data were first tested for normal distribution by the Kolmogorov–Smirnov normality test. Normally distributed data were then statistically analyzed using one-way ANOVA with GraphPad Prism (version 8.0.0 for Windows, GraphPad Software, San Diego, CA, USA, www.graphpad.com, accessed on 21 April 2022) followed by Tukey’s honestly significant difference calculations. Data were presented as the mean of each group ± the standard error of the mean (SEM), and the significance was declared at *p* < 0.05.

## 3. Results

### 3.1. HPLC Analysis of AvS

**Figure 1:**
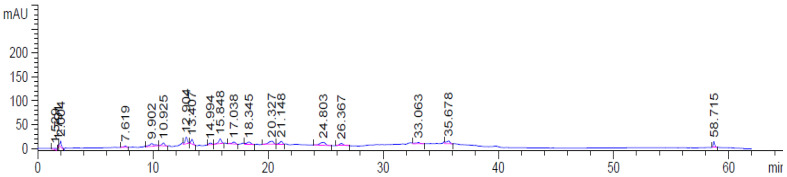
The HPLC chromatogram of the AvS ethanolic extract shows different peaks representing the phenolic compounds of the seeds.

Table 2 and Figure 1 show the results of HPLC analysis for AvS. HPLC chromatograms for the AvS revealed the presence of numerous phenolic compounds (gallic acid, protocatechuic acid, *p*-hydroxybenzoic acid, catechin, caffeic acid, vanillic acid, ferulic acid, sinapic acid, *p*-coumaric acid, rosmarinic acid, apigenin-7-glucoside, cinnamic acid, quercetin, and chrysin). Protocatechuic acid was the major phenolic compound (35.51 μg/g), followed by catechin (30.80 μg/g), and caffeic acid (12.44 μg/g).

### 3.2. AvS Ameliorated Liver Function Deteriorated by CsA 

Table 3 shows the effect of treatment with AvS and/or CsA on liver damage parameters. Rats injected with CsA (CsA group) exhibited significant increases in the serum activities of liver function enzymes (AST, ALT, ALP, γ-GTP) and levels of total, direct and indirect bilirubin. However, they came with a significantly decreased albumin level compared to the control (Cnt) group. However, in animals co-treated with AvS and CsA (AvS + CsA group), the activities and levels of these parameters were partially restored but were still significantly higher than those in Cnt and AvS groups. No significant difference was noticed between AvS and Cnt groups.

### 3.3. AvS restored Hepatic Oxidant and Antioxidant Status Disturbed by CsA

Table 4 shows the influence of AvS and/or CsA on the levels of the lipid peroxidation marker MDA and the activities of SOD, CAT, and GPx antioxidant enzymes in the livers. Administration of AvS alone (AvS group) did not exhibit any significant changes in the levels and activities of these markers compared to the control group. However, the injection of CsA significantly disturbed the redox balance. This was revealed by the elevation of MDA and reduction of SOD, CAT, and GPx in the CsA group relative to the two control groups (Cnt and AvS). On the other hand, animals co-treated with AvS and CsA restored the redox balance, as elicited by the significantly decreased MDA levels and the significantly increased activities of the antioxidant enzymes in the liver of rats co-treated with AvS and CsA, compared to those treated with CsA alone.

### 3.4. AvS inhibited Proapoptotic ER Stress Induced by CsA

**Figure 2:**
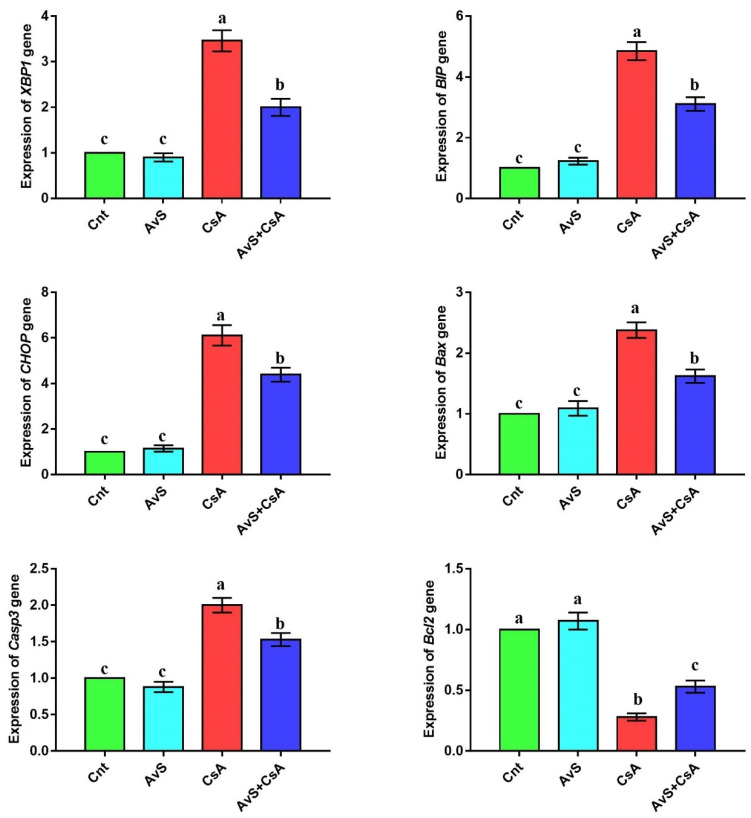
Influence of treatment with AvS and/or CsA on the hepatic expression of ER stress-related genes (*XBP1*, *BIP*, and *CHOP*) and apoptosis-related genes (*Bax*, *Casp3*, and *Bcl2*), as revealed by real-time PCR. Data were presented as fold change mean ± SEM (*n* = 8/group). Columns with different letters (a–c) are significantly different at *p* < 0.05. All groups were compared to each other.

The altered expression of ER stress-related genes (*XBP1*, *BIP*, and *CHOP*) and apoptosis-related genes (*Bax*, *Casp3*, and *Bcl2*) was detected in the liver by real-time PCR after treatment with AvS and/or CsA (Figure 2 and Supplementary File). No significant difference in the expression of these genes was noticed between the two control groups (Cnt and AvS). In contrast, the CsA group showed significantly upregulated hepatic expression of *XBP1*, *BIP*, *CHOP*, *Bax*, and *Casp3* genes and significantly downregulated expression of *Bcl2* compared to the two control groups. Interestingly, animals co-treated with AvS and CsA showed significantly lower expression of *XBP1*, *BIP*, *CHOP*, *Bax*, and *Casp3* genes and significantly higher expression of *Bcl2* than animals treated with CsA alone.

### 3.5. AvS Alleviated DNA Damage Caused by CsA

**Figure 3:**
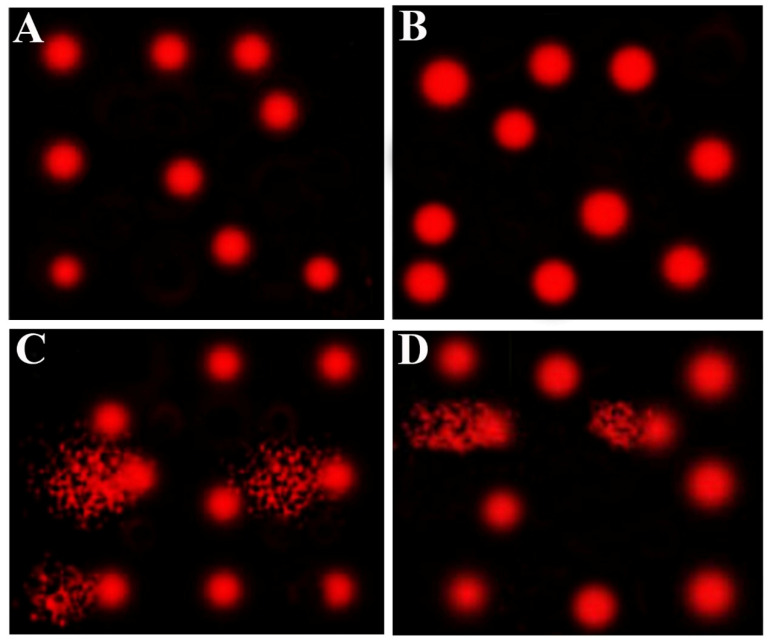
Representative comet assay images show the influence of treatment with AvS and/or CsA on hepatic cells’ DNA damage. The assay was carried out in 3 independent experiments. (**A**) Cnt, (**B**) AvS, (**C**) CsA, (**D**) AvS + CsA. In (**C**,**D**), the fragmented patches refer to the comet tail which contains degraded DNA, while the front red circle refers to the intact DNA.

Table 5 and Figure 3 show the effect of AvS and/or CsA on DNA damage of hepatic cells, as revealed by the comet assay. The CsA group elicited a significant increase in DNA damage, as revealed by higher tail length, tail DNA%, and tail moment, compared to the two control (Cnt and AvS) groups. This increased DNA damage was significantly diminished in the co-treated group (AvS + CsA) but did not reach the normal levels as in the two control groups. No significant changes were observed between Cnt and AvS groups.

### 3.6. AvS Relieved Hepatic Damage Score Induced by CsA

**Figure 4:**
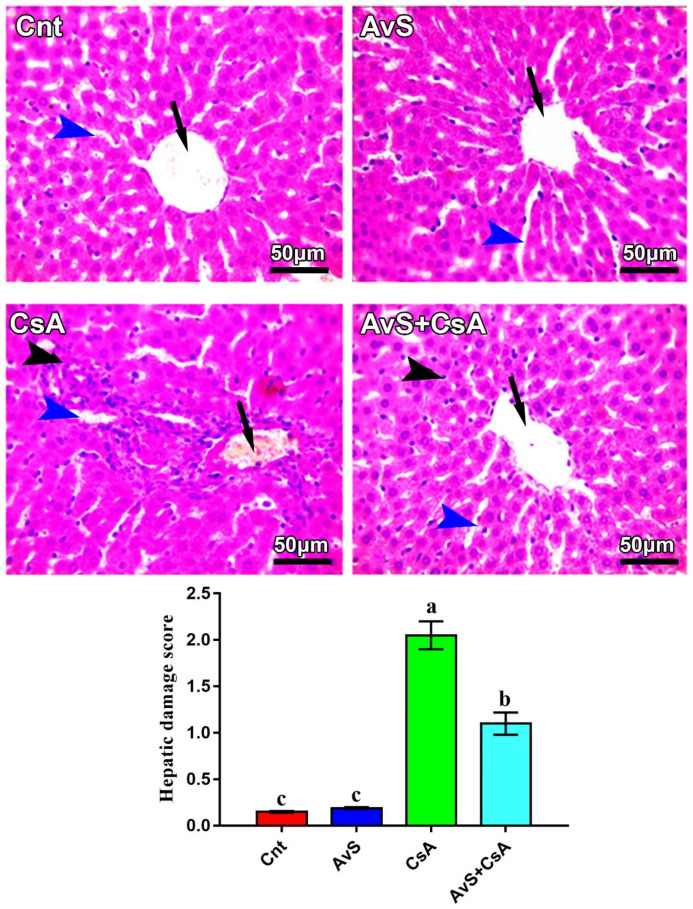
Photomicrographs of hepatic sections stained with H&E. Arrows refer to central veins, blue arrowheads refer to hepatic sinusoids, and black arrowheads refer to mononuclear cell infiltration. Scale bars: 50 μm. The bar graph shows the hepatic damage score, and values are the mean ± SEM (*n* = 3/group). Columns carrying different letters (a–c) are significantly different at *p* < 0.05.

Figure 4 shows changes in liver histology following treatment with AvS and/or CsA. Both Cnt and AvS groups showed normal hepatic histology, with radially arranged hepatic cords around the central vein (arrows) and normal-sized hepatic sinusoids (blue arrowheads). However, the CsA group showed congested central and portal veins (arrow), notable mononuclear cell infiltration, especially around the central and portal vein (black arrowhead) and dilated hepatic sinusoids (blue arrowheads). On the other hand, the liver of the co-treated group (AvS + CsA) elicited notable improvement in liver histology, with only a mild degree of mononuclear cell infiltration and slightly dilated sinusoids. The hepatic damage score was significantly higher in the CsA group and was significantly decreased in the AvS and CsA co-treated group.

## 4. Discussion

Recent evidence confirmed that CsA-induced nephrotoxicity involved the activation of ER stress [17,18]. So far, no data were available in the literature addressing whether CsA-induced hepatotoxicity could also be mediated by ER stress activation. We recently found an ameliorative effect for AvS, which had potent antioxidant properties, against CsA-induced nephrotoxicity [10]. To the best of our knowledge, this is the first study to report that CsA-induced hepatotoxicity involves activation of proapoptotic ER stress, as revealed by the upregulation of ER stress-related genes (*XBP1*, *BIP*, and *CHOP*) and apoptosis-related genes (*Bax* and *Casp3*), and the downregulation of the anti-apoptotic gene *Bcl2* in livers of CsA-treated rats. The other novel results of this study included ameliorative effects of AvS against CsA-induced hepatotoxicity, as evidenced by reduced liver damage parameters (ALT, AST, ALP, γ-GTP, and bilirubin), restored redox balance (reduction of MDA and elevation of antioxidant enzymes), inhibited ER stress and DNA damage, and improved liver histology.

CsA-induced hepatotoxicity is associated with functional and structural disturbances in the liver. Functionally, we reported that CsA increased serum activities and levels of liver damage parameters (ALT, AST, ALP, γ-GTP, and bilirubin), and decreased albumin expression. This disrupted liver function indicates the induction of hepatotoxicity by CsA. Consistent with our findings, several studies reported similar elevations of these parameters in animals treated with CsA [6,7,8,9,11,12,13,14,39]. Patients injected with CsA a long time after kidney transplantation had high levels of AST, ALT, and bilirubin, and additionally suffered from cholestasis due to the inhibition of the ATP-dependent transport of bilirubin and bile salts from liver cells to hepatic canaliculi [5,40]. This can also explain the elevation of bilirubin in the serum of CsA-treated rats.

Structurally, we found that the injection of CsA induced dilation of hepatic sinusoids, mononuclear inflammatory cell infiltration, and congestion of portal and central veins. Similar pathological lesions were also noticed by other studies following the treatment of animals with CsA [6,7,11,12,14,41]. However, unlike these studies, we did not find degenerative changes in the hepatocytes or focal hepatocyte necrosis. This could be attributed to the short duration of the treatment with CsA (7 days). Indeed, other studies that used the same treatment duration did not report vacuolar degeneration or necrosis [19].

Molecularly, oxidative stress, inflammation, and apoptosis are the main three mechanisms involved in CsA-mediated hepatotoxicity. In the first mechanism (oxidative stress), CsA, which is highly lipophilic, is immediately bound to cell membranes and triggers lipid oxidation (as revealed by high levels of MDA) that causes the overproduction of free radicals, especially from mitochondria, and inhibits activities of endogenous cellular antioxidant enzymes such as SOD, CAT, and GPx [6,7,9,10,11,12,14,15]. In support of this, we also found high hepatic levels of MDA and low activities of SOD, CAT, and GPx in rats treated with CsA. High levels of MDA indicate lipid peroxidation which causes oxidative damage to various cell components and suppresses the endogenous antioxidant defense system (SOD, GPx, CAT) [42,43,44,45,46]. As the mitochondrion is the main organelle that generates reactive oxygen species (ROS), CsA could interrupt mitochondrial oxidative phosphorylation in hepatocytes (which contain abundant mitochondria), leading to extensive ROS release and redox imbalance [11,12,47].

In the second mechanism (inflammation), we found mononuclear inflammatory cell infiltration (mainly neutrophils and macrophages) in the liver of rats injected with CsA. In agreement, other studies reported the aggregation of neutrophils and macrophages in the vicinity of portal regions following CsA administration [7,11]. These inflammatory cells induce the release of pro-inflammatory cytokines which participate in tissue damage. Although CsA is a potent immunosuppressive drug, it upregulated the expression of inflammatory cytokines TNFα, Il1β, NFκB, and COX2 in the liver of rats [6,11,12,13]. This differential effect of CsA on cytokines could be attributed to CsA ability to selectively inhibit IL2 (rather than other cytokines such as TNFα and Il1β), which subsequently hinders T-cell replication and immune response [48].

In terms of the third mechanism (apoptosis), we reported that CsA activated the apoptotic pathway through the upregulation of the apoptotic *Bax* and *Casp3* genes and the downregulation of the antiapoptotic *Bcl2* gene in the liver. A similar apoptotic effect was reported for CsA on animal livers by other studies [6,11,12,13]. CsA can trigger apoptosis in renal cells via many pathways such as mitochondrial dysfunction and ROS generation [49]. Additionally, CsA induces ROS generation which damages mitochondrial membranes, allowing the release of cytochrome c from mitochondria into the cytoplasm in a process that activates Casp3 [50]. Further confirmation for the induction of apoptosis by CsA was obtained from the comet assay, which showed increased DNA damage and fragmentation in liver cells of rats treated with CsA alone. Similar DNA damage was noticed for CsA on the renal cells and sperms [10,16].

In the present study, we provided a novel mechanism by which CsA could mediate its hepatotoxic effect. For the first time, we reported that injection of CsA resulted in significant upregulation of ER stress-related genes (*XBP1*, *BIP*, and *CHOP*) in the liver. Similarly, CsA-induced nephrotoxicity has been shown to involve the activation of ER stress [17]. Indeed, patients who underwent kidney transplantations and were injected with CsA showed increased mRNA levels of ER stress markers in their kidneys [18,51]. Taken together, activation of ER stress could mediate both the nephrotoxicity and hepatotoxicity caused by CsA. It is well-known that CsA inhibits the activity of the holoenzyme calcineurin through undergoing binding with cyclophilin. The latter acts as a chaperone, a protein that prevents the misfolding of other proteins, and so its inhibition by CsA could lead to protein misfolding through activation of ER stress [52]. To ensure survival, the cell achieves adaptive mechanisms such as the induction of proper protein folding through the modulation of *XBP1* and *BIP* expression [53]. If these adaptive mechanisms failed, the expression of *CHOP* and its downstream target proapoptotic gene *Bax* would be increased [18]. Similarly, our results revealed the upregulation of *CHOP* and its downstream targets *Bax* and *Casp3*, along with downregulated *Bcl2* expression in the livers of CsA-treated rats. This infers failure of the adaptive mechanism and activation of the proapoptotic pathway. Targeting ER stress by chaperones was progressively considered a promising strategy to mitigate liver diseases [54]. In parallel, it has been recently shown that CsA could induce the proapoptotic ER stress pathway, as revealed by upregulation of *XBP1*, *BIP*, *CHOP*, *Bax*, and *Casp3* expression in renal tubular cells, and this effect was relieved by appropriate chaperones [18].

Unlike rats treated with CsA alone, rats co-treated with CsA and AvS elicited distinguished improvements as indicated by the reduction in liver damage parameters, lipid peroxidation, ER stress, apoptosis, inflammation, and the elevation of antioxidant status. This implies that AvS has potent antioxidant, anti-inflammatory, anti-ER stress, and anti-apoptotic effects against CsA-induced hepatotoxicity. In general, these results imply that consumption of AvS had hepatoprotective potential against liver damage triggered by CsA in rats. A similar antioxidant-mediated hepatoprotective effect for avocado pulp extract was reported against liver damage induced by potassium dichromate [29] or CCL_4_ [30]. The potent antioxidant potential of AvS might be due to its contents of health-promoting constituents such as triterpenes, hydrocinnamic acid, catechin, and epicatechin [24,55]. Our HPLC data revealed the presence of numerous phenolic compounds, with abundant amounts of protocatechuic acid, catechin, and caffeic acid. On the other hand, the extract prepared from the avocado pulp contained abundant amounts of catechin, caffeic acid, ferulic acid, sinapic acid, cinnamic acid, and apigenin [29]. Comparing the phenolic profile of AvS with that of avocado pulp, the seeds had higher protocatechuic acid than the pulp. Protocatechuic acid has many pharmacological effects that are owed to its antioxidant activities [56]. This acid is more active in lipid and aqueous media and, therefore, it could be used in the food industry or pharmacological preparation as a natural antioxidant. Other main compounds found in AvS are catechin and caffeic acid which also have potent antioxidant activities [57,58]. Overall, phenolic compounds of AvS have been reported as potent antioxidants which reduce lipid peroxidation and minimize tissue damage-dependent oxidative stress [59].

In the present study, the co-administration of AvS and CsA decreased inflammation, as indicated by the reduced mononuclear cell infiltration in the liver. This implies that AvS has anti-inflammatory potential, in addition to its antioxidant properties. In support of this conclusion, avocado and its seeds significantly decreased cytokines and ROS release [60,61,62]. Moreover, avocado pulp had a potent anti-inflammatory effect through the downregulation of inflammation-related genes such as *NfkB*, *IL6*, and *TNFα* in rat liver [29]. Interestingly, we found that AvS also downregulated the expression of ER stress-related genes (*XBP1*, *BIP*, and *CHOP*). Recently, it has been reported that avocado oil extract can protect against ototoxicity through the activation of autophagy [63]. Moreover, the activation of autophagy has been shown to restrict inflammation and mitigate ER stress [64]. Further studies are required to check whether mitigation of ER stress by AvS is associated with the induction of autophagy. AvS are also safe on healthy cells as they have no genotoxic effect [24]. A large amount of AvS is produced as waste during avocado processing, and their uncontrollable removal may lead to environmental pollution. Therefore, it could be better to utilize AvS or their active ingredients as natural food additives, and as adjuvants to relieve CsA-induced hepatotoxicity.

## 5. Conclusions

To the best of our knowledge, this is the first study to report that AvS affords protection against CsA-induced hepatotoxicity through their antioxidants, anti-inflammatory, anti-ER stress, and antiapoptotic properties. This health-promoting effect could be due to the inhibition of ER stress markers (*XBP1*, *BIP*, and *CHOP*), apoptotic markers (*Bax*, *Casp3*), and inflammatory cytokines. Therefore, these markers may be medicinal targets in CsA-induced hepatotoxicity, especially for patients treated with CsA for a long time. However, further investigations are needed to unveil the precise mode of AvS action and to verify whether AvS treatment is effective and safe for humans.

## Figures and Tables

**Table 1:** Primers used for real-time PCR.

Gene	Forward Primer	Reverse Primer
*XBP1*	AAACAGAGTAGCAGCGCAGACTGC	GGATCTCTAAAACTAGAGGCTTGGTG
*BIP*	CTGGGTACATTTGATCTGACTGG	GCATCCTGGTGGCTTTCCAGCCATTC
*CHOP*	AGGAGAGAGAAACCGGTCCAA	GGACACTGTCTCAAAGGCGA
*Bax*	ACACCTGAGCTGACCTTG	AGCCCATGATGGTTCTGATC
*Casp3*	GGTATTGAGACAGACAGTGG	CATGGGATCTGTTTCTTTGC
*Bcl2*	ATCGCTCTGTGGATGACTGAGTAC	AGAGACAGCCAGGAGAAATCAAAC
*GAPDH*	CAACTCCCTCAAGATTGTCAGCAA	GGCATGGACTGTGGTCATGA

**Table 2:** Phenolic profile of avocado seeds as detected by HPLC.

Compound	Retention Time	Concentration (μg/g)
Gallic acid	3.98	1.87
Protocatechuic acid	7.79	35.51
Gentisic acid	12.41	Not detected
*p*-hydroxybenzoic acid	14.59	7.08
Catechin	16.69	30.80
Caffeic acid	18.94	12.44
Syringic acid	21.43	Not detected
Vanillic acid	22.94	0.53
Ferulic acid	33.43	5.57
Sinapic acid	35.42	4.07
*p*-Coumaric acid	37.75	0.75
Rutin	39.19	Not detected
Rosmarinic acid	42.62	0.97
Apigenin-7-glucoside	49.37	1.14
Cinnamic acid	53.96	0.65
Quercetin	55.99	0.38
Apigenin	58.66	Not detected
Kaempferol	59.97	Not detected
Chrysin	61.22	1.90

**Table 3:** Effect of AvS treatment on liver damage parameters in CsA-induced hepatotoxicity in rats.

Parameters	Cnt	AvS	CsA	AvS + CsA
ALT (U/L)	34.23 ± 1.74 ^c^	32.53 ± 2.15 ^c^	90.33 ± 5.64 ^a^	61.46 ± 2.79 ^b^
AST (U/L)	79.20 ± 5.81 ^c^	72.05 ± 5.36 ^c^	208.52 ± 13.15 ^a^	119.27 ± 8.53 ^b^
ALP (U/L)	159.36 ± 7.03 ^c^	141.92 ± 7.44 ^c^	260.39 ± 18.82 ^a^	207.09 ± 14.13 ^b^
γ-GTP (U/L)	9.15 ± 0.52 ^c^	8.36 ± 0.47 ^c^	28.25 ± 1.42 ^a^	19.18 ± 0.84 ^b^
Albumin (g/dL)	2.95 ± 0.15 ^a^	2.67 ± 0.16 ^a^	1.70 ± 0.10 ^b^	2.39 ± 0.13 ^a^
Total bilirubin (mg/dL)	0.35 ± 0.01 ^c^	0.36 ± 0.01 ^c^	1.12 ± 0.05 ^a^	0.62 ± 0.03 ^b^
Direct bilirubin (mg/dL)	0.11 ± 0.007 ^c^	0.08 ± 0.002 ^c^	0.32 ± 0.01 ^a^	0.13 ± 0.008 ^b^
Indirect bilirubin (mg/dL)	0.24 ± 0.01 ^c^	0.28 ± 0.01 ^c^	0.80 ± 0.04 ^a^	0.49 ± 0.02 ^b^

Values are presented as mean ± SEM (*n* = 8 rats per group). Data with dissimilar letters [^a^ (the highest)–^c^ (the lowest)] in the same row are significantly different at *p* ≤ 0.05. All groups were compared to each other. Cnt: control group; AvS: avocado seed group; CsA: cyclosporine A group; AvS + CsA: avocado seed and cyclosporine A group.

**Table 4:** Effects of AvS on hepatic oxidant and antioxidant status in rats with CsA-induced hepatotoxicity.

Parameters	Cnt	AvS	CsA	AvS + CsA
MDA (nmol/g tissue)	39.00 ± 1.39 ^c^	36.27 ± 1.54 ^c^	146.83 ± 7.62 ^a^	81.44 ± 4.50 ^b^
SOD (U/g tissue)	35.5 ± 1.56 ^a^	37.11 ± 1.64 ^a^	8.93 ± 0.42 ^c^	17.19 ± 0.56 ^b^
CAT (U/g tissue)	54.66 ± 1.27 ^a^	55.17 ± 1.40 ^a^	18.83 ± 0.77 ^c^	35.28 ± 1.03 ^b^
GPx (U/g tissue)	28.75 ± 0.92 ^a^	31.83 ± 1.04 ^a^	13.16 ± 0.42 ^c^	22.83 ± 0.70 ^b^

Values are presented as mean ± SEM (*n* = 8 rats per group). Data with dissimilar letters [^a^ (the highest)–^c^ (the lowest)] in the same row are significantly different at *p* ≤ 0.05. All groups were compared to each other. Cnt: control group; AvS: avocado seed group; CsA: cyclosporine A group; AvS + CsA: avocado seed and cyclosporine A group.

**Table 5:** Effect of AvS ameliorated hepatic cells’ DNA damage induced by CsA, as detected by the comet assay.

Parameters	Cnt	AvS	CsA	AvS + CsA
Tailed %	3	4.5	20	12
Untailed %	97	95.5	80	88
Tail length (µm)	1.75 ± 0.11 ^c^	2.00 ± 0.14 ^c^	6.14 ± 0.35 ^a^	4.01 ± 0.28 ^b^
Tail DNA%	1.68	1.82	5.35	3.19
Tail moment *	2.94	3.64	32.85	12.79

Values are presented as mean ± SEM (*n* = 8 rats per group). Data with dissimilar letters [^a^ (the highest)–^c^ (the lowest)] in the same row are significantly different at *p* ≤ 0.05. All groups were compared to each other. * Tail moment is the tail DNA (%) X tail length (µm). Cnt: control group; AvS: avocado seed group; CsA: cyclosporine A group; AvS + CsA: avocado seed and cyclosporine A group.

## Data Availability

The data supporting the present findings are contained within the manuscript.

## Supplementary Materials

The following supporting information can be downloaded at: https://www.mdpi.com/article/10.3390/molecules27227859/s1, Supplementary file shows raw data of real time PCR.

## Sample Availability

Samples of the compounds are not available from the authors.
