# Supplementary material for: Avocado Seeds-Mediated Alleviation of Cyclosporine A-Induced Hepatotoxicity Involves the Inhibition of Oxidative Stress and Proapoptotic Endoplasmic Reticulum Stress
Source: Molecules. 2022 Nov 14;27(22):7859. doi: 10.3390/molecules27227859 (PMC9698978; doi:10.3390/molecules27227859)
Supplement: Supplementary file 1 [file molecules-27-07859-s001.zip › molecules-1739795-supplementary.pdf]

Suppl file shows raw data of real time PCR

1) XBP1 gene

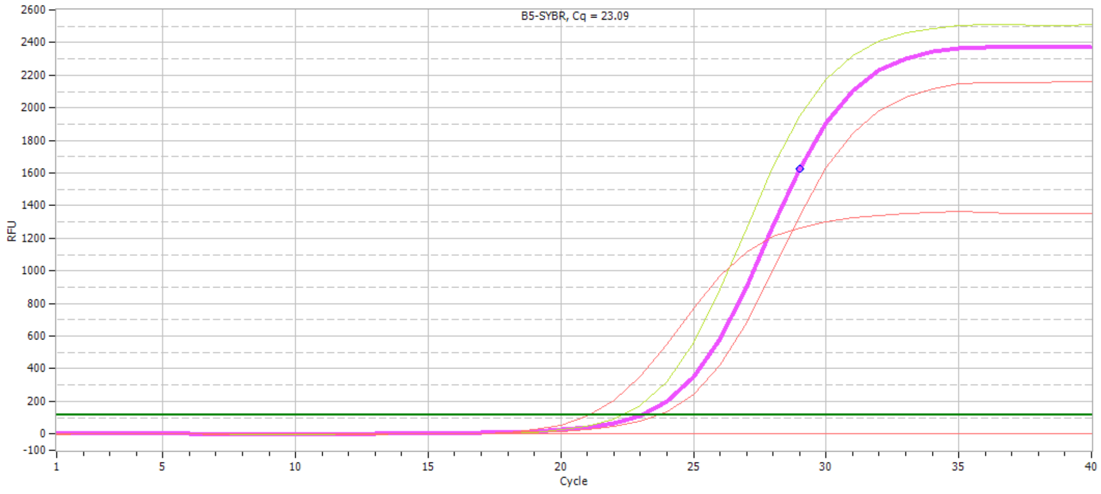

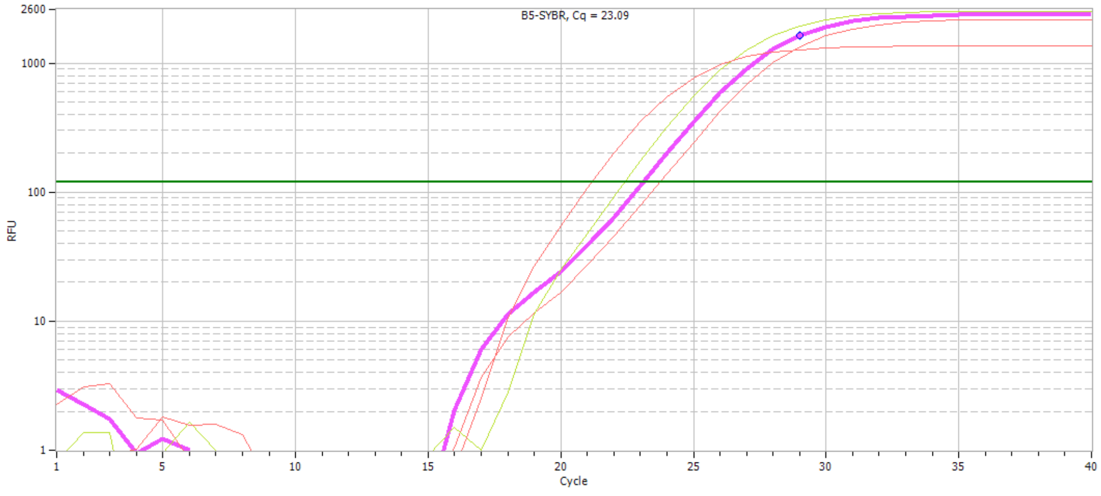

Linear (upper) and log (lower) views of the amplification curves representing the Ct values of *XBP1*.

Calculation of fold change

Group	*XBP1* Aver CT	Delta Ct	Delta delta Ct	Fold change	SEM
Cnt	23.09	-6.04	0.00	1.00	0
AvS	23.69	-5.97	0.07	0.95	0.06
CsA	21.12	-6.02	0.02	0.99	0.1
AvS+ CsA	22.33	-5.83	0.21	0.86	0.05

2) BIP gene

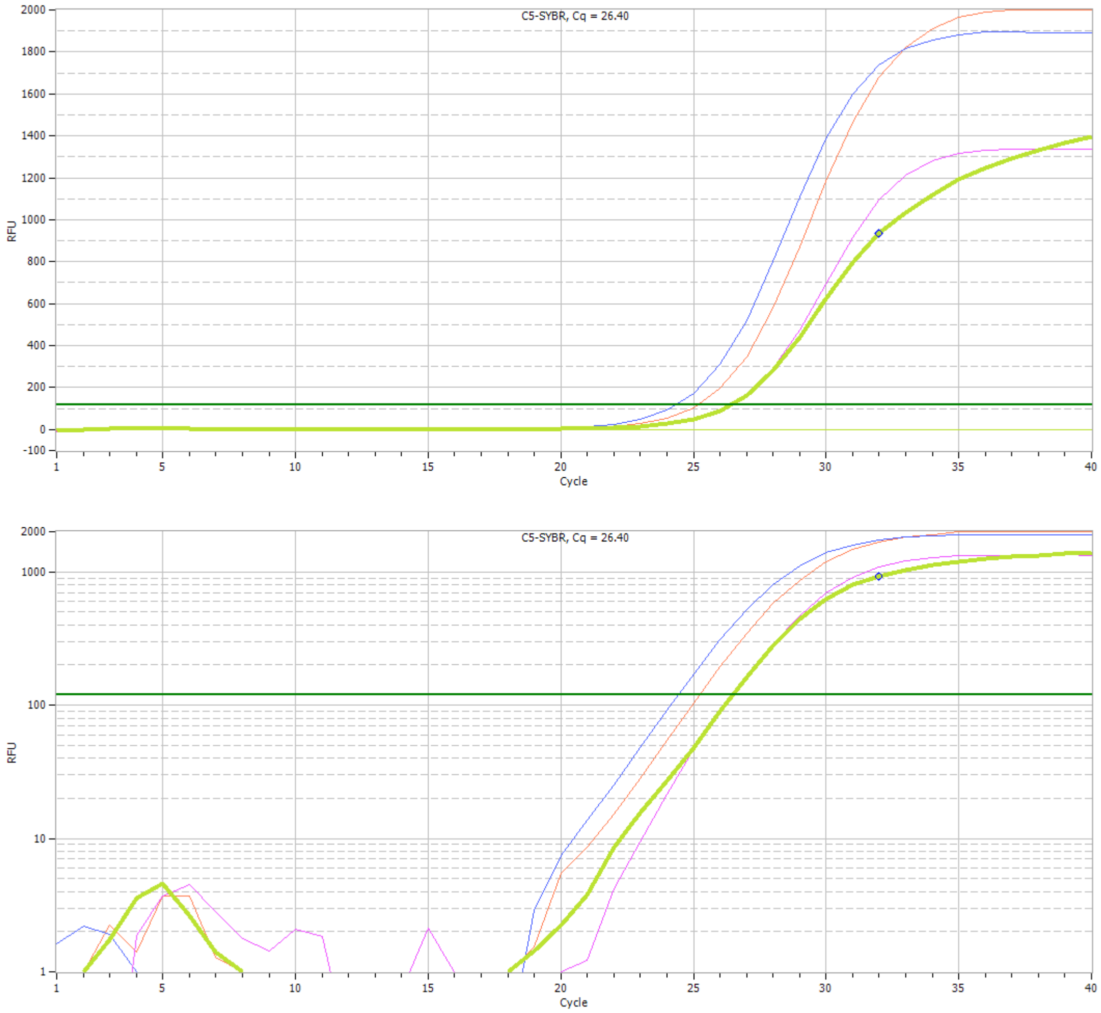

Linear (upper) and log (lower) views of the amplification curves representing the Ct values of *BIP*.

Calculation of fold change

Group	*BIP* Aver CT	Delta Ct	Delta delta Ct	Fold change	SEM
Cnt	26.4	2.01	0.00	1.00	0
AvS	26.37	1.71	-0.30	1.23	0.11
CsA	24.35	-0.27	-2.28	4.86	0.3
AvS+ CsA	25.16	0.37	-1.64	3.12	0.22

3) CHOP gene

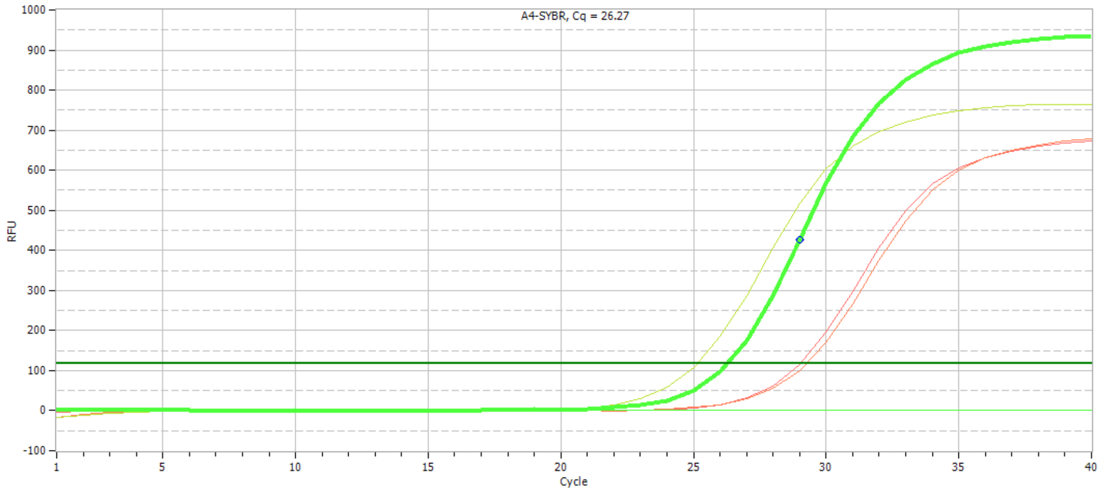

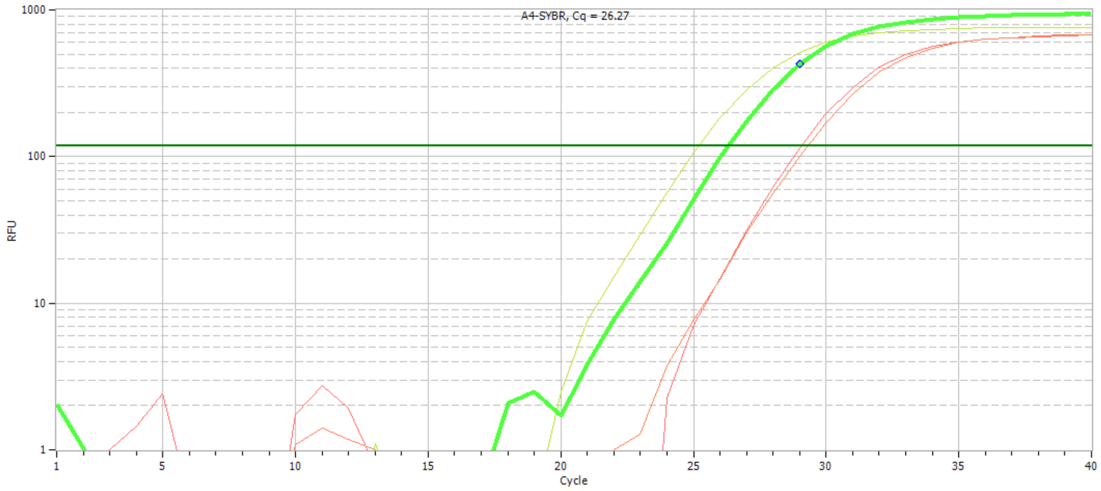

Linear (upper) and log (lower) views of the amplification curves representing the Ct values of *CHOP*.

Calculation of fold change

Group	*CHOP* Aver CT	Delta Ct	Delta delta Ct	Fold change	SEM
Cnt	29.06	4.67	0.00	1.00	0
AvS	29.27	4.47	-0.20	1.15	0.14
CsA	25.16	2.06	-2.61	6.11	0.45
AvS+ CsA	26.27	2.54	-2.13	4.38	0.31

4) Bax gene

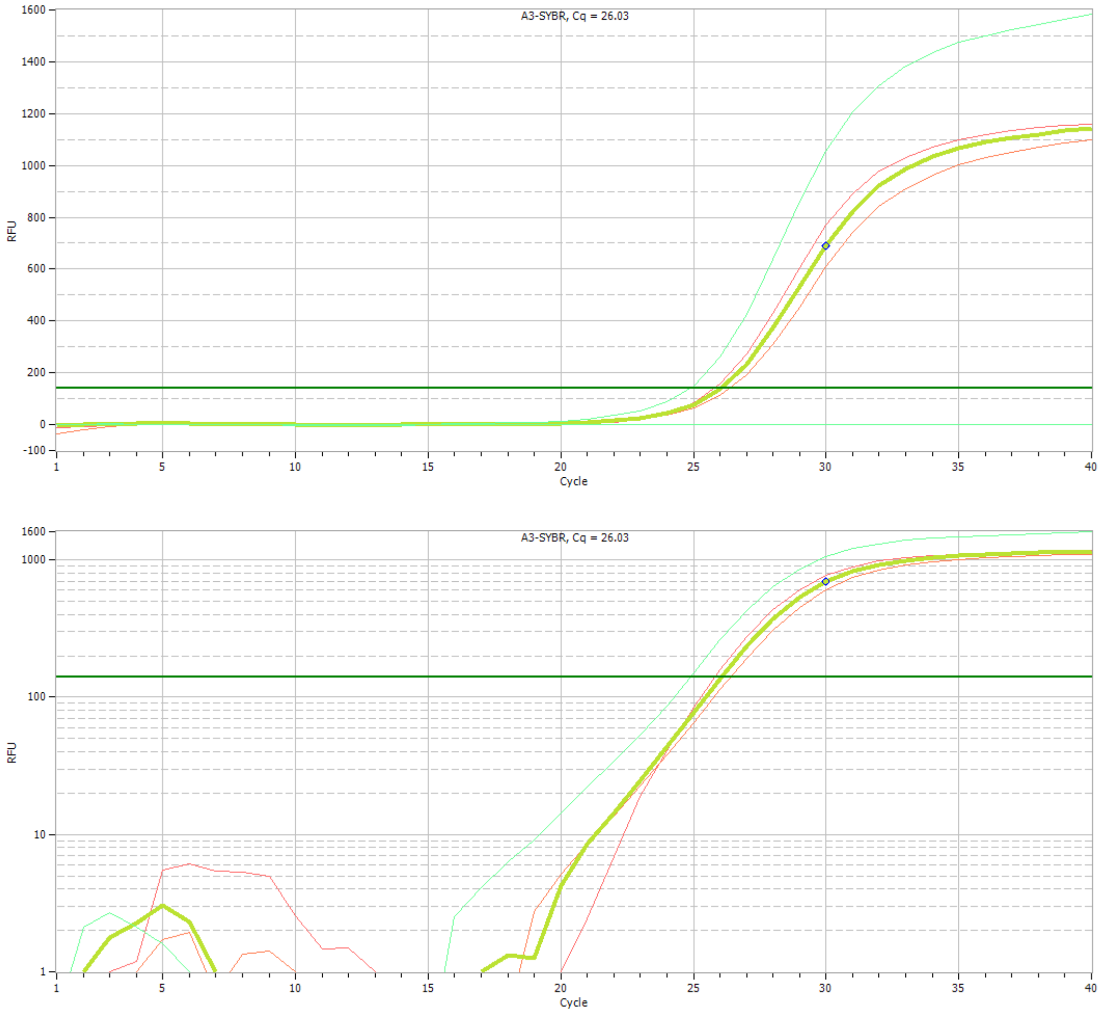

Linear (upper) and log (lower) views of the amplification curves representing the Ct values of *Bax*.

Calculation of fold change

Group	*Bax* Aver CT	Delta Ct	Delta delta Ct	Fold change	SEM
Cnt	26.33	1.94	0.00	1.00	0
AvS	26.03	1.81	-0.13	1.09	0.12
CsA	24.82	0.69	-1.25	2.38	0.13
AvS+ CsA	25.74	1.24	-0.70	1.62	0.11

5) Casp3 gene

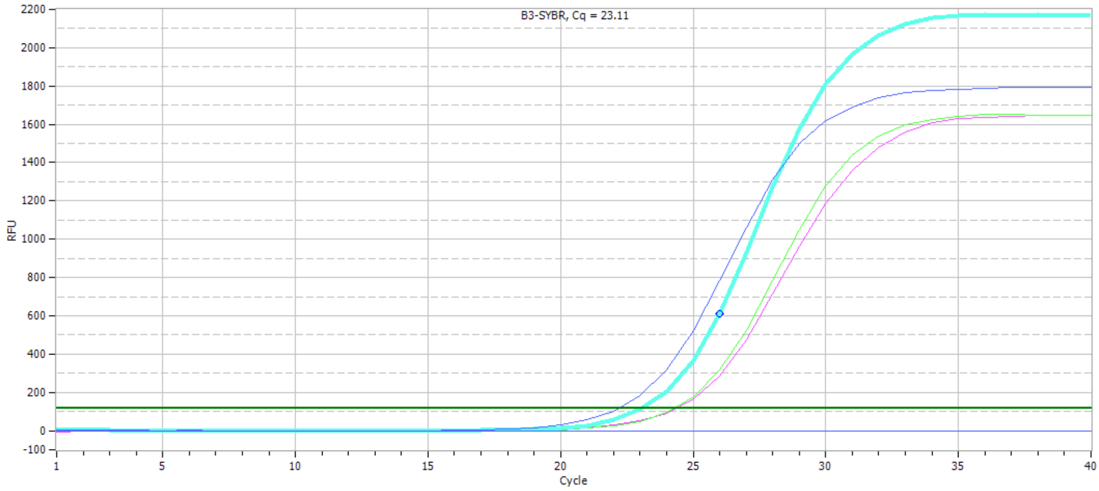

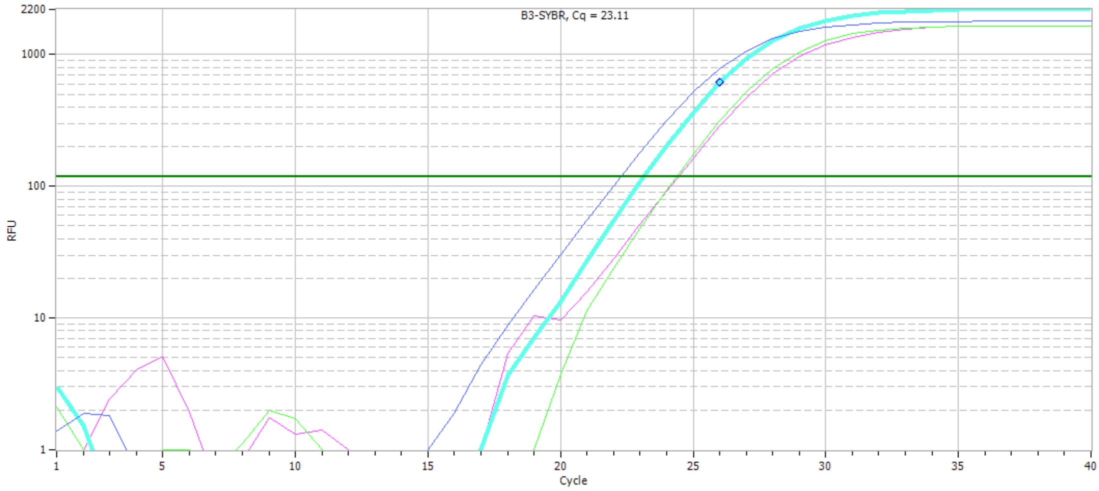

Linear (upper) and log (lower) views of the amplification curves representing the Ct values of *Casp3*.

Calculation of fold change

Group	*Casp3* Aver CT	Delta Ct	Delta delta Ct	Fold change	SEM
Cnt	24.31	-0.08	0.00	1.00	0
AvS	24.39	0.10	0.18	0.88	0.07
CsA	22.23	-1.08	-1.00	2.00	0.1
AvS+ CsA	23.11	-0.69	-0.61	1.53	0.09

6) Bcl2 gene

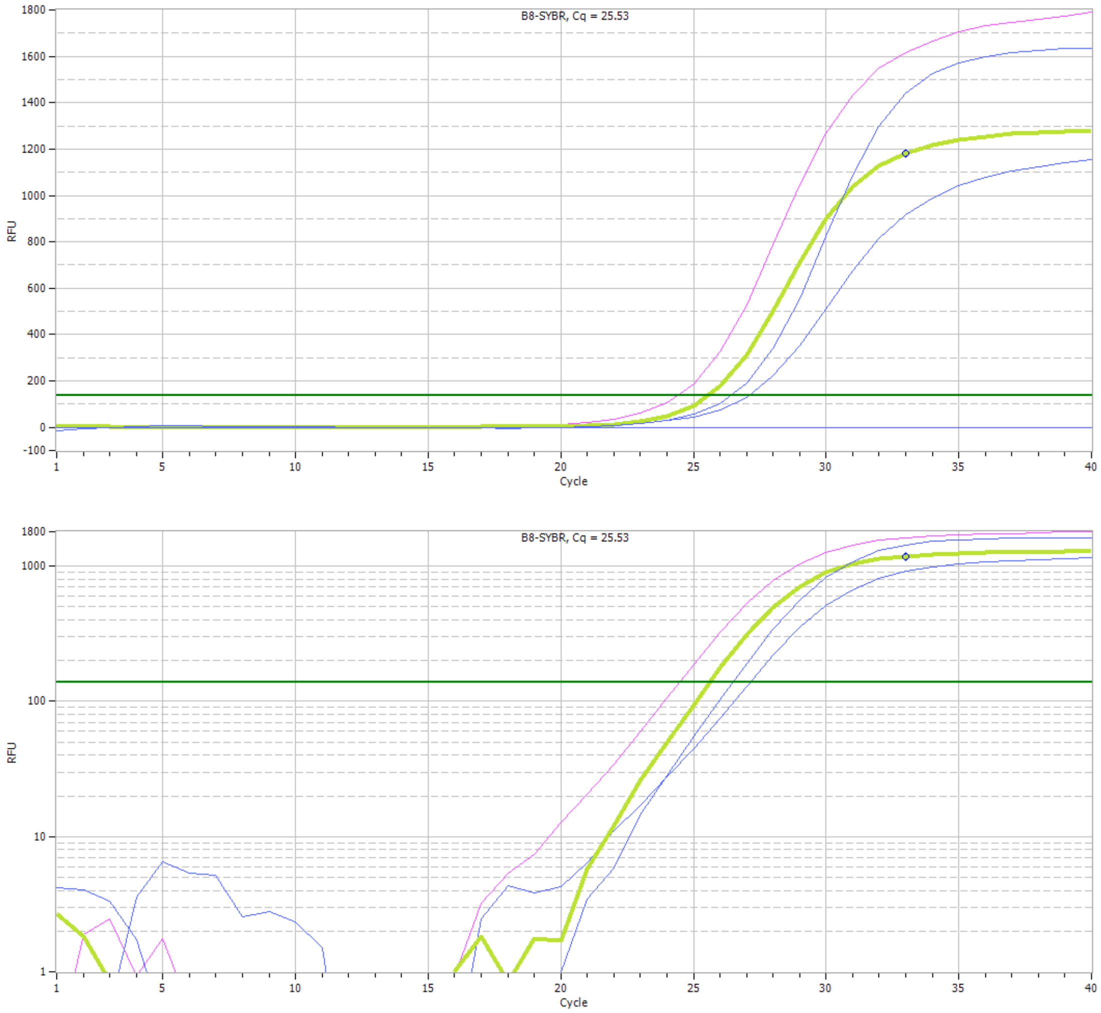

Linear (upper) and log (lower) views of the amplification curves representing the Ct values of *Bcl2*.

Calculation of fold change

Group	*Bcl2* Aver CT	Delta Ct	Delta delta Ct	Fold change	SEM
Cnt	25.53	1.14	0.00	1.00	0
AvS	24.4	1.04	-0.10	1.07	0.07
CsA	27.1	2.97	1.83	0.28	0.03
AvS+ CsA	26.41	2.05	0.91	0.53	0.03
